# Supplementary material for: Cigarette smoke extract (CSE) reduces expression of functional TRPV4 channels in primary human bronchial epithelial cells differentiated at an air liquid interface (ALI) in vitro
Source: Arch Toxicol. 2026 Jun 13;100(10):4695–704. doi: 10.1007/s00204-026-04475-z (PMC13593732; doi:10.1007/s00204-026-04475-z)
Supplement: Supplementary file 1 [file 204_2026_4475_MOESM1_ESM.pdf]

Cigarette Smoke Extract (CSE) reduces expression of functional TRPV4 channels in primary human bronchial epithelial cells differentiated at an Air Liquid Interface (ALI) in vitro

Isabel Müller · Philipp Alt · Thomas Gudermann · Martina Kiefmann · Alexander Dietrich

Walther Straub Institute of Pharmacology and Toxicology, Member of the German Center for Lung Research (DZL), Medical Faculty, LMU-Munich, Munich, Germany

Supporting information:

Table S1 List of human donors of primary human bronchial epithelial cells (pHBECs) differentiated at an ALI:

Number	Sex	Age	Race	Health condition	Smoker	Alcohol use
18TL190281	Male	75	B	Non- diseased	No	No
21TL347555	Male	55	C	Non- diseased	No	No
23TL240134	Male	47	H	Non- diseased	No	No

Table S2: List of oligonucleotide sequences, used for qRT-PCR.

Gene of interest	forward sequence	reverse sequence
TRPV4	GTCCACACAGAGATGGCAGTT	GCAGAAAGGAAGGTCTGGGG
ARK1C1	CTGCAGAGGTTCCTAAAA	CCTGCTCCTCATTATTGTA
AKR1B10	GTGTTGCAATCCTCTCAT	GGACATGAGTGGAGGTAGT
PIR	GTAAGGATGGTGTGACAGTT	GTCCACCCTTTAGGGATA
CYP1B1	GCCACTATCACTGACATCT	CAGGATACCTGGTGAAGA
CYP1A1	CTTGGACCTCTTTGGAGCTG	CGAAGGAAGAGTGTCGGAAG
NQO1	AAGGACATCACAGGTAAACT	GAACTGGAATATCACAAGGT
ALDH3A1	CCTGACTACATCCTCTGTG	CCCGTAGAACTCTTTCAGT

A

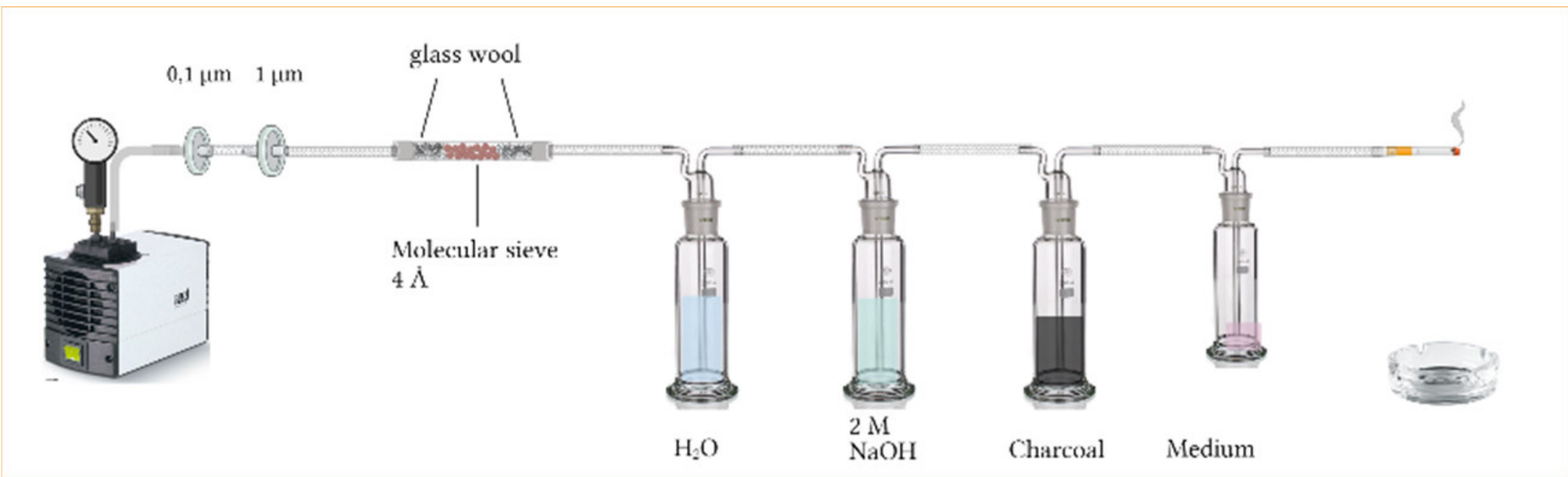

B

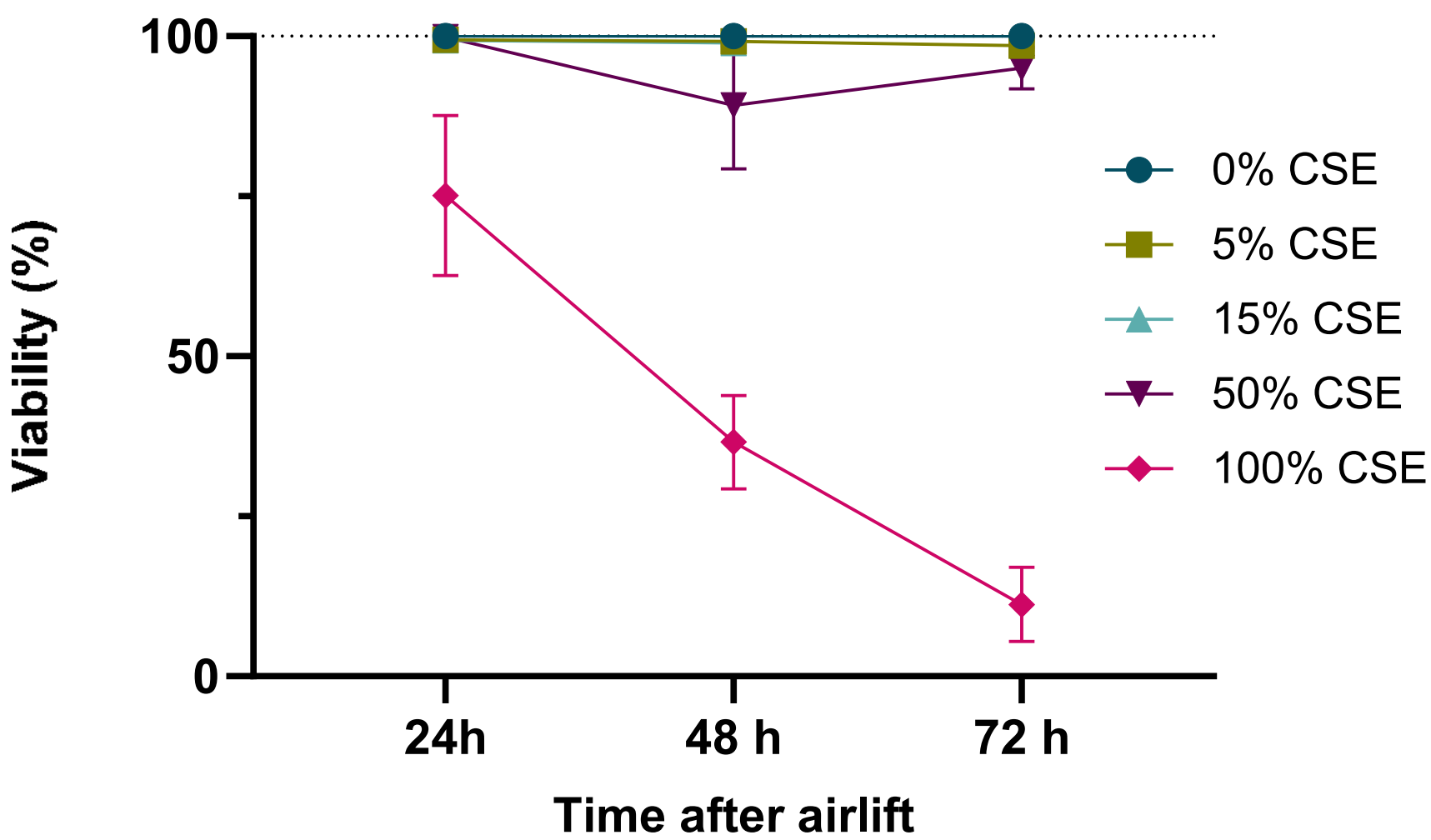

Fig. S1 Production of Cigarette Smoke Extract (CSE) and viability of pHBECs after application of different concentrations of CSE. **A** Experimental setup for the production of 100% CSE. **B** CSE was applied chronically directly after the air lift to pHBECs in the indicated concentrations and cell lysis was quantified at the specified time points of differentation at an ALI by a lactate dehydrogenase (LDH) assay.

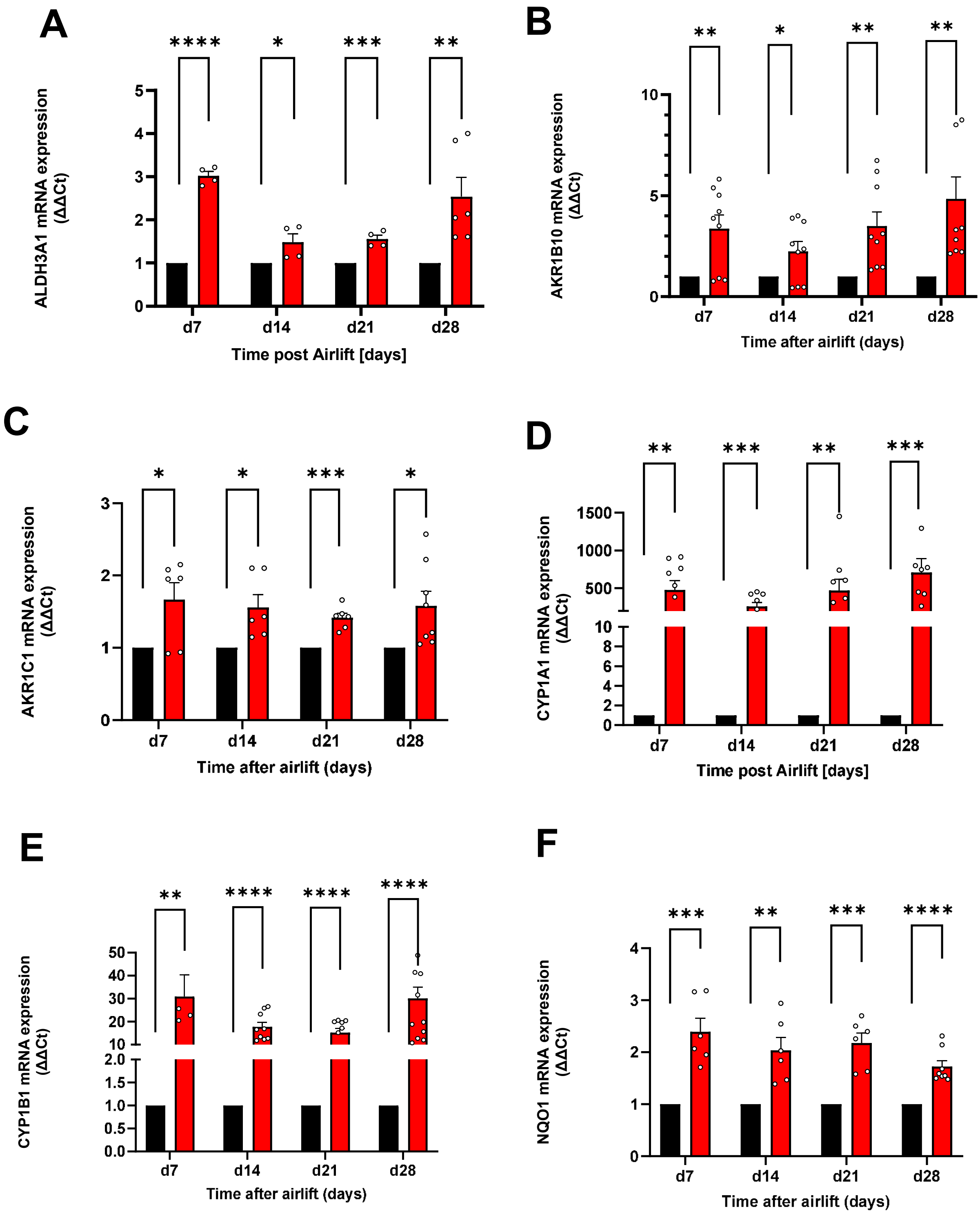

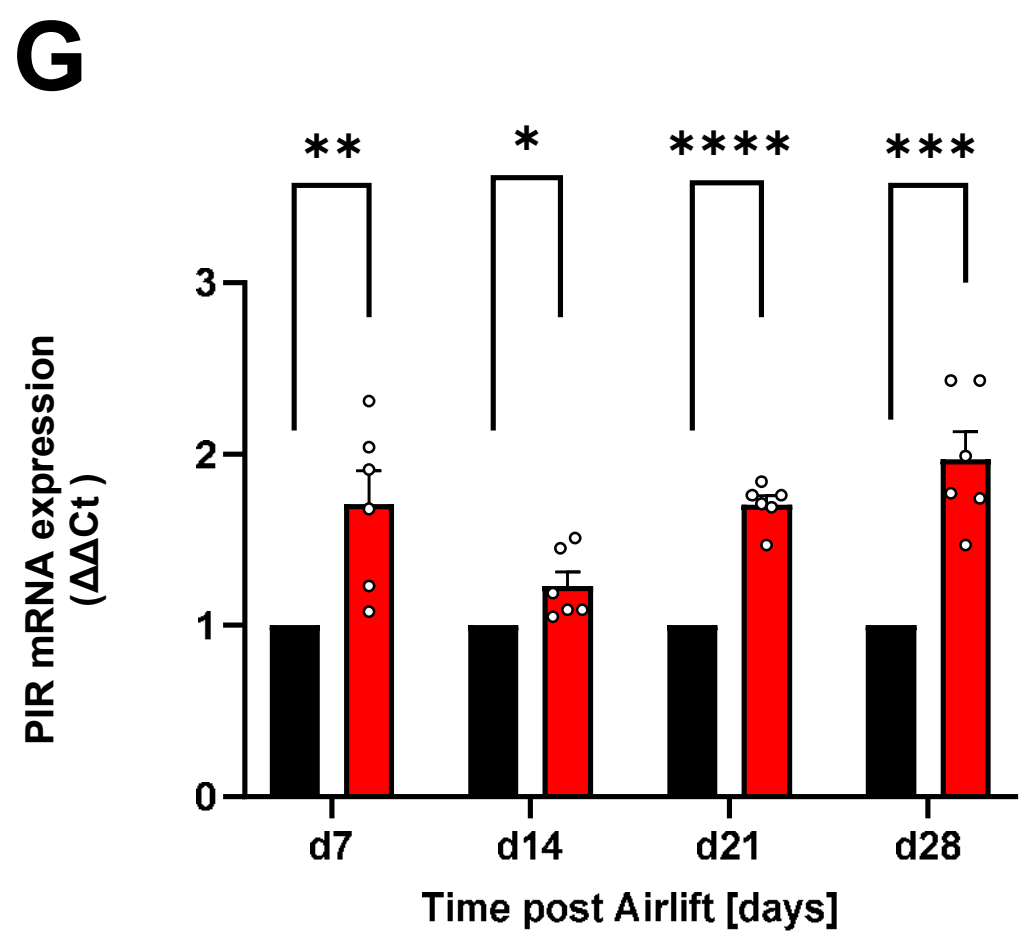

Fig. S2 Up-regulation of mRNA expression from smoke regulated genes (SERGs) after chronic application of 5% CSE (red bars) in pHBECs from the indicated time points of differentiation at the ALI in comparison to untreated controls (black bars). Values are shown as ΔΔCt to β-actin as housekeeping gene. **A** Aldehyde dehydrogenase 3 family member A1 (ALDH3A1), **B** Aldo-keto-reductase family 1 member B10 (AKR1B10), **C** Aldo-keto-reductase family 1 member C1 (AKR1C1), **D** Cytochrome P450 1A1 (Cyp1A1), **E** Cytochrome P450 1B1 (Cyp1B1), **F** NAD(P)H quinone dehydrogenase 1 (NQO1), **G** Pirin (PIR).

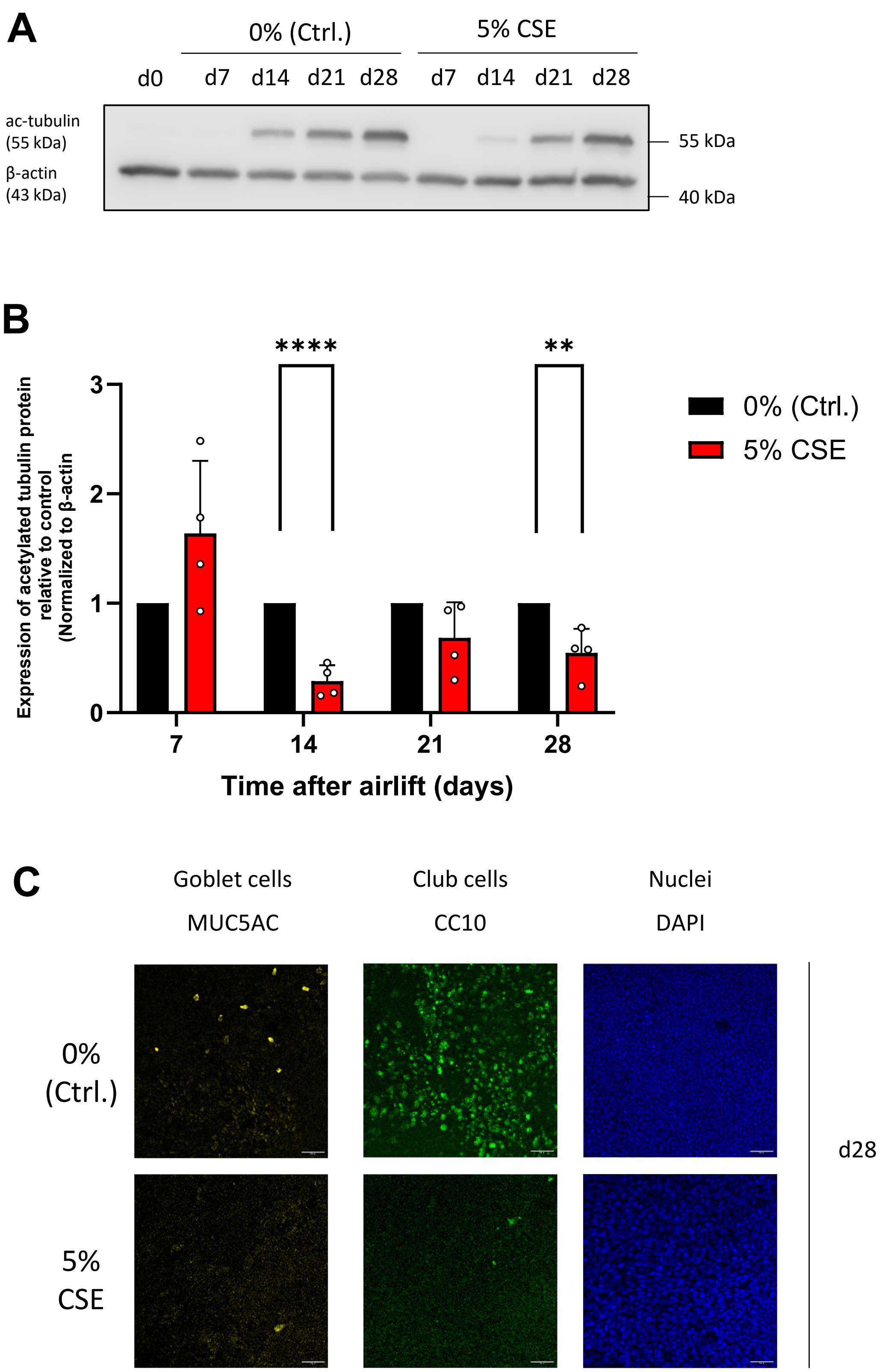

Fig. S3 Acetylated tubulin, goblet and club cells are reduced in differentiated pHBECs after application of CSE. A Representative Western Blot of pHBECs lysates preincubatd with (5% CSE) or without CSE (0% (Ctrl.) from the indicated time points of differentiation incubated with a specific antibody directed against acetylated tubulin. Beta actin was used as loading control. B Quantification of acetylated tubulin in pHEBCs preincubated with (5% CSE, red bars) or without CSE (0% (Ctrl.), black bars). Values are shown relative to controls and normalized to β-actin expression. Data represent means + SD of at least 4 experiments. Significance between means was analyzed using multiple unpaired Student's t-test and is indicated as *** for $p<0.001$ and ** for $p<0.01$. C Representative confocal images of the differentiated pHBECs with (5% CSE) or without chronic CSE exposure (0%, Ctrl.). Goblet cells were identified by staining with fluorescent coupled antibodies directed against mucin 5AC (yellow, left panels) and club cells by staining with fluorescent coupled antibodies directed against Clara Cell 10-kDa protein (CC10) (green, middle panels). All cell nuclei were stained with DAPI (blue, right panels).

A

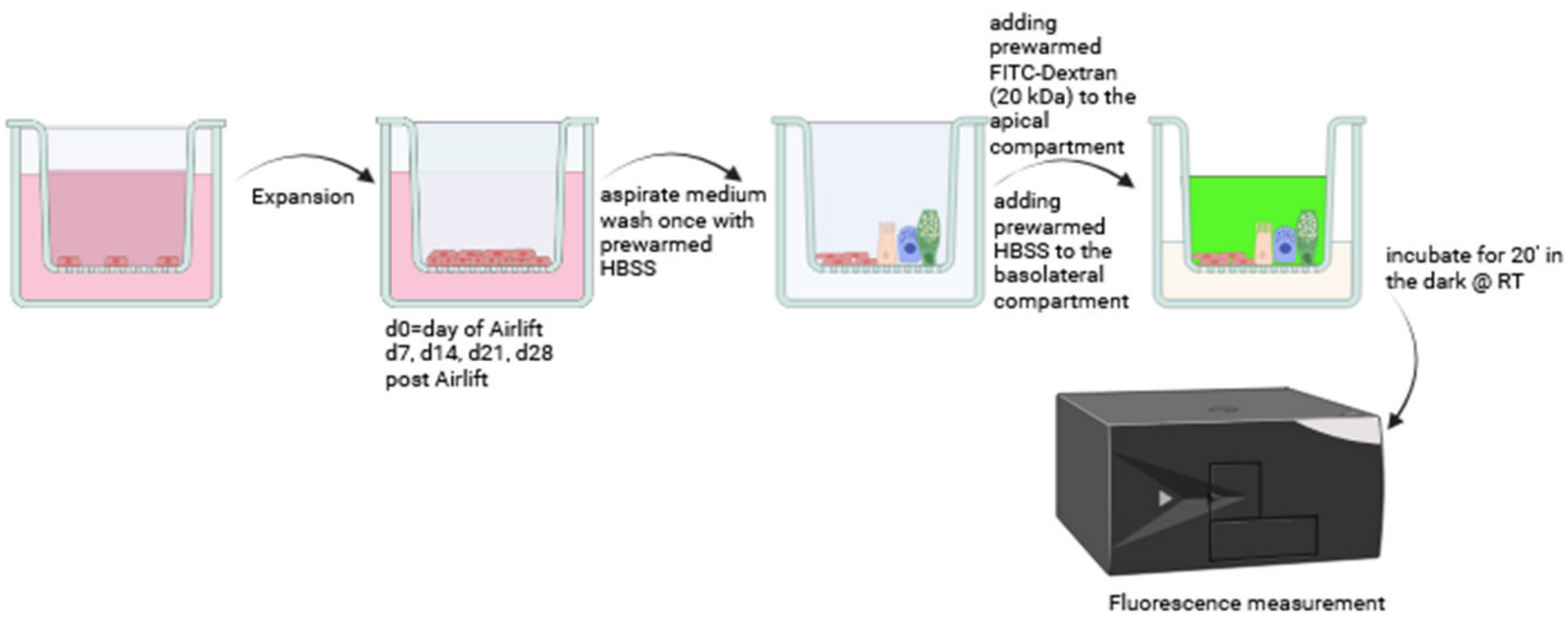

B

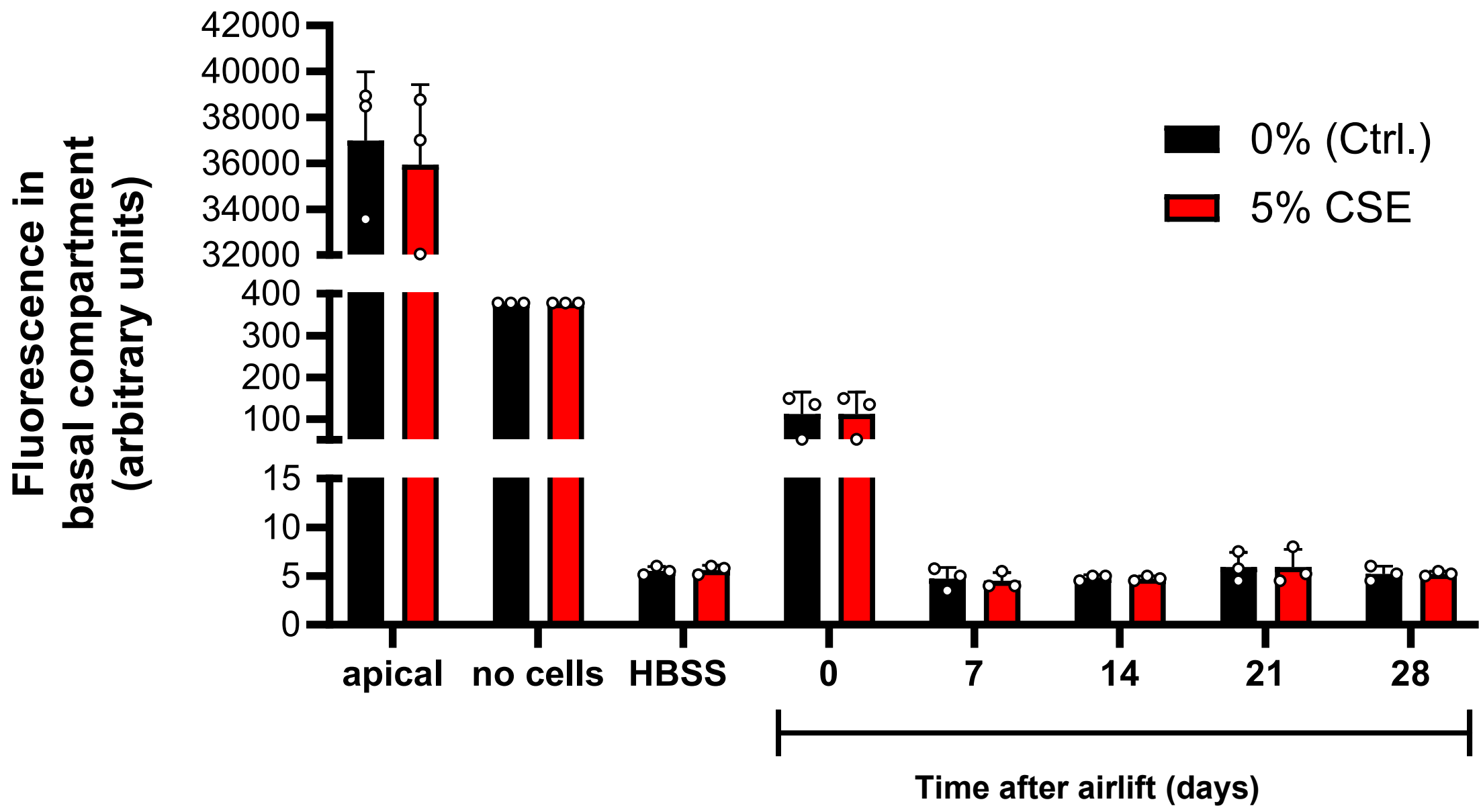

Fig. S4 Cell permability is not changed in pHBECs differentiated at the ALI incubated with or without CSE. **A** Experimental set up of the permeability assay. After final differentiation of pHBECs a suspesion of FITC-dextrane particles were added to the apical compartment and fluorescence from the basolateral compartment was quantified in a photometer after incubation of 20 min. in the dark. **B** Quantification of cell permeability of pHBECs differentiated at the ALI incubated with (5% CSE, red bars) or without CSE (0% (Ctrl.), black bars). Fluorescence was measured in the apical (apical) or basal compartments in HBSS (HBSS), in an ALI model with no cells (no cells) or with pHBECs differentiated for 0 to 28 days.

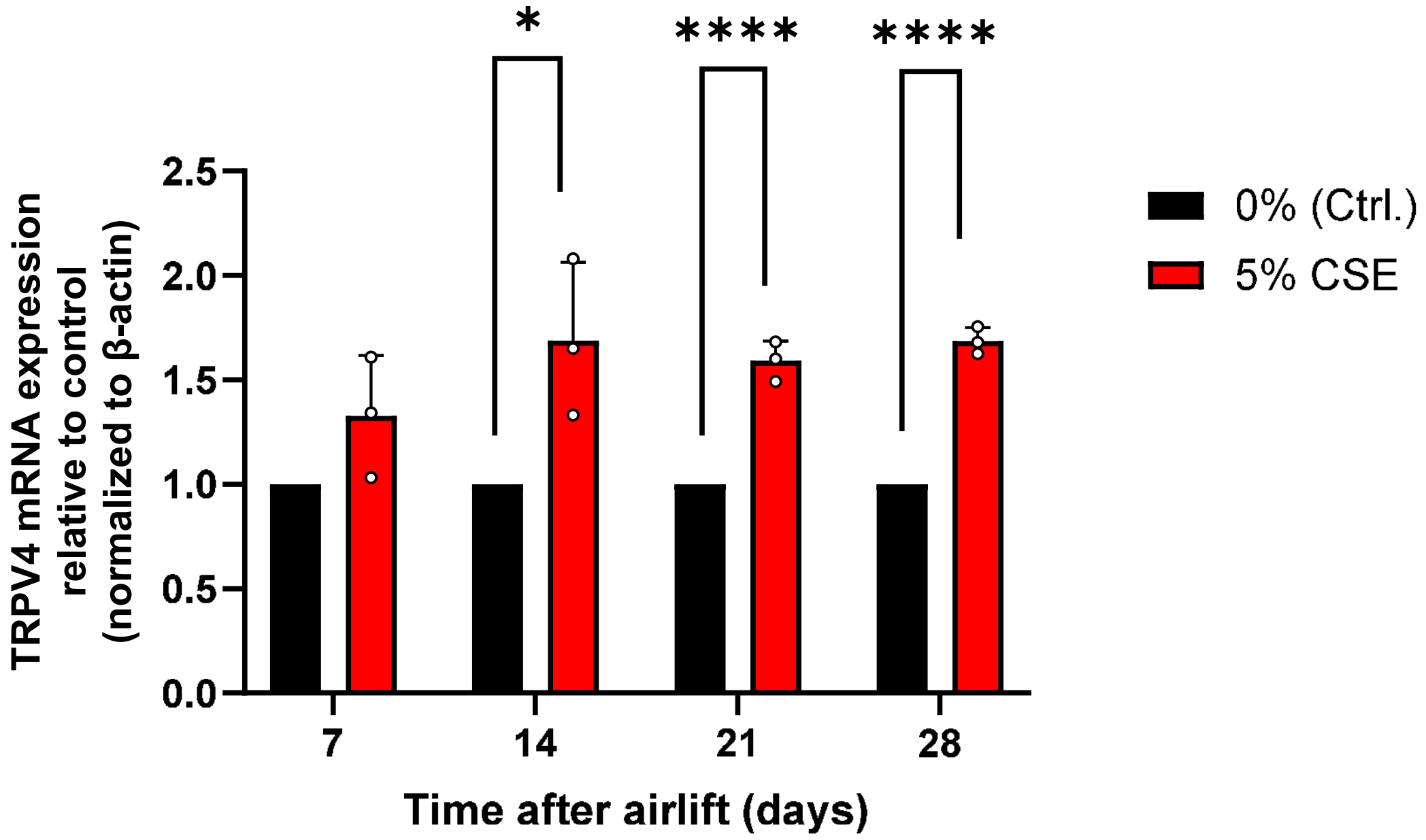

Fig. S5 Expression of TRPV4 mRNA is increased in pHBECs differentiated at an ALI by CSE **A** Quantification of TRPV4 mRNA expression in pHBECs differentaiated for the indicated time points with (5% CSE, red bar) or without CSE (0% (Ctrl.), black bars) by quantitative reverse transcription (qRT) PCR. Values are shown relative to controls and normalized to β-actin expression. Data represent means + SD from at least three experiments. Significance between means was analyzed using two-way ANOVA and is indicated as *** for $p<0.001$ and * for $p<0.05$.
